# Plants oxidative response to nanoplastic

**DOI:** 10.3389/fpls.2022.1027608

**Published:** 2022-10-20

**Authors:** Anna Ekner-Grzyb, Anna Duka, Tomasz Grzyb, Isabel Lopes, Jagna Chmielowska-Bąk

**Affiliations:** ^1^ Department of Cell Biology, Institute of Experimental Biology, Faculty of Biology, School of Natural Sciences, Adam Mickiewicz University, Poznań, Poland; ^2^ Department of Plant Ecophysiology, Institute of Experimental Biology, Faculty of Biology, School of Natural Sciences, Adam Mickiewicz University, Poznań, Poland; ^3^ Department of Mycology and Plant Resistance, Vasily Nazarovich Karazin (VN) Karazin Kharkiv National University, Kharkiv, Ukraine; ^4^ Department of Rare Earths, Faculty of Chemistry, Adam Mickiewicz University, Poznań, Poland; ^5^ Department of Biology & Centre for Environmental and Marine Studies (CESAM), University of Aveiro, Aveiro, Portugal

**Keywords:** polystyrene (PS), oxidative stress, reactive oxygen species, lipid peroxidation, antioxidant enzymes

## Abstract

Pollution of the environment with plastic is an important concern of the modern world. It is estimated that annually over 350 million tonnes of this material are produced, wherein, despite the recycling methods, a significant part is deposited in the environment. The plastic has been detected in the industrial areas, as well as farmlands and gardens in many world regions. Larger plastic pieces degraded in time into smaller pieces including microplastic (MP) and nanoplastic particles (NP). Nanoplastic is suggested to pose the most serious danger as due to the small size, it is effectively taken up from the environment by the biota and transported within the organisms. An increasing number of reports show that NP exert toxic effects also on plants. One of the most common plant response to abiotic stress factors is the accumulation of reactive oxygen species (ROS). On the one hand, these molecules are engaged in cellular signalling and regulation of genes expression. On the other hand, ROS in excess lead to oxidation and damage of various cellular compounds. This article reviews the impact of NP on plants, with special emphasis on the oxidative response.

## Graphical Abstract

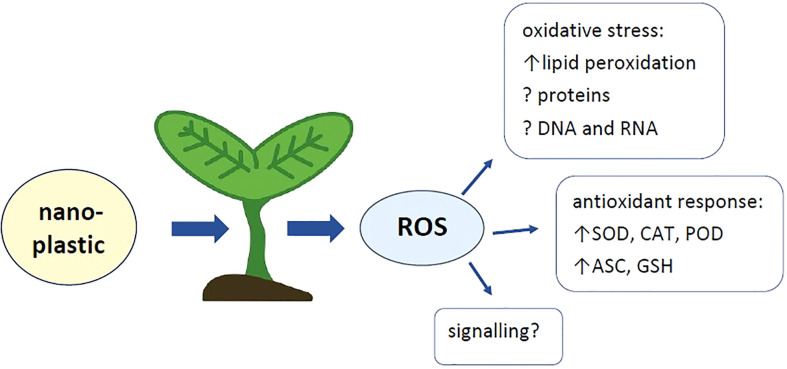

## Introduction

Plastics in each form are composed of polymeric material in most cases industrially artificially synthesized, whether in the bulk form or as micro- and nanoparticles (MP and NP, respectively). Microplastic (MP) is considered plastic particles with sizes greater than 1 µm to 1 mm, but this definition is still not standardized, and other size ranges can be found in many reports or publications (Horton et al., 2017) Plastic fragments smaller than 1 µm are considered nanoplastic (NP) (Hartmann et al., 2019). Usually, plastics are synthetic organic compounds manufactured from fossil fuel-based chemicals like petroleum and natural gas. Natural polymers such as silk or rubber are also sources of plastics, but only synthetic plastics are non-biodegradable. The history of plastics starts with the beginning of the 20^th^ century and the invention of Bakelite (Crespy et al., 2008). Since then, plastics have become one of the primary synthetic materials used. The evolution of production methods and many advantages of plastics over other materials such as glass, paper, wood, metals, or ceramics caused that in 2022, approximately 450-500 million tons of plastic will be produced (Geyer et al., 2017; Law and Narayan, 2022; Zhao et al., 2022).

**Figure 1:**
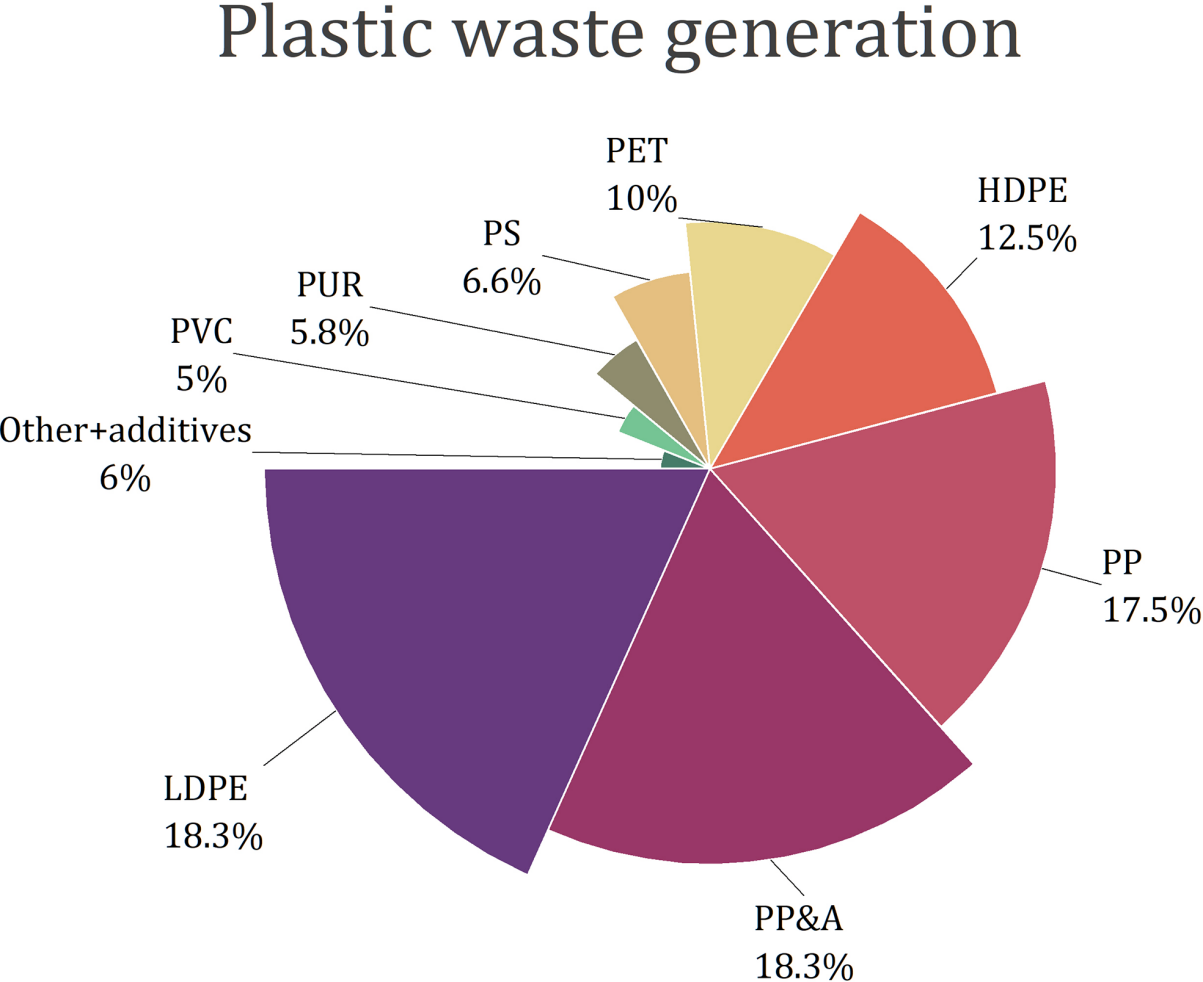
Composition of plastic waste generated globally in 2015 (Geyer et al., 2017).

The most frequently used plastics are composed of polyethylene (PE), polypropylene (PP), polystyrene (PS), polyvinyl chloride (PVC), and polyethylene terephthalate (PET) (Geyer et al., 2017; Schwarz et al., 2019; Zhao et al., 2022; **Figure 1**). Other types of plastic materials are polyurethanes (PUR) and polyester, polyamide, and acrylic (PP&A) fibres (Geyer et al., 2017). Polyethylene is produced in two forms: low-density PE (LDPE) and high-density PE (HDPE). Around 60% of solid plastic waste contains LDPE, HDPE and PP (Butler et al., 2011). However, plastic waste composition strongly depends on the place and varies between landfills, rivers, oceans or soil (Gwada et al., 2019; Schwarz et al., 2019; Antonopoulos et al., 2021). For example, in freshwaters and oceans, PE is the main component of plastics, followed by PP and PS - these three polymers together make up from 92.2 to 95.8% of waste (Schwarz et al., 2019).

The degradation of plastics is a long and complex process (Lucas et al., 2008). Estimates show that plastic materials require 250-1000 years to completely degrade, depending on chemical composition and structure (Chamas et al., 2020). It was one of the advantages of plastics responsible for their wide use. However, since plastics are considered a challenging environmental hazard after decomposition to MP and NP, their chemical stability and resistance become a problem because they decompose after a long period of time in the environment. Plastics in the environment undergo biodegradation or non-biodegradation processes (Lucas et al., 2008). Thermal degradation, photo- and oxidative degradation or hydrolysis are all examples of non-biodegradation (Lucas et al., 2008; Yee et al., 2021). Plastics, in the presence of sunlight and water, are naturally decomposed into simpler structures - oligomers and monomers (Lucas et al., 2008; Lambert and Wagner, 2016; Yee et al., 2021). Additionally, the chemical structure of polymers and their fragments also changes in the presence of water, oxygen and light, forming esters, ketones, alcohols, or even acids when the environment is acidic, which makes plastics more hydrophilic (Lucas et al., 2008; Chamas et al., 2020). The processes mentioned above occur only on the MP and NP surface, whereas the higher surface-to-volume ratio of NP makes them especially reactive. Some species of bacteria and other microorganisms such as *Aspergillus tubingensis*, *Pestalotiopsis microspore* or *Zalerion maritimum* can decompose plastics through enzyme-supported reactions (Lucas et al., 2008; Yuan et al., 2020). In the natural environment, both processes occur parallelly, i.e. plastic decomposition products, such as simple organic compounds, are assimilated by microbial cells, which may also affect the health of these organisms (Ng et al., 2018). Microorganisms are also responsible for accelerating plastic degradation, e.g. by covering the surface of plastics or infiltrating the porous plastic structures (Lucas et al., 2008; Gaur et al., 2020).

Plastics contain chemical additives such as remaining solvents, catalysts, dyes, plasticizers and many others (Kiran et al., 2022). During the production of plastics, different chemical compounds are added to colorize or improve the resistance of polymers to the degradation (Kiran et al., 2022). Toxic or potentially toxic substances, such as Bisphenol A, are used to manufacture various plastics (Narevski et al., 2021; Kiran et al., 2022). Many plasticizers, such as 1,2-benzenedicarboxylic acid, chlorinated paraffins or formaldehyde are neurotoxic or carcinogenic (Kiran et al., 2022). Plastics can also be a vector of various chemicals, such as persistent organic pollutants (POPs), e.g. 1,1,1-Trichloro-2,2-bis[p-chlorophenyl]-ethane (DDT), polycyclic aromatic hydrocarbons, hexachlorobenzene, polychlorinated dibenzofurans and many other chemicals produced by the industry (Nizzetto et al., 2012; Antunes et al., 2013; Kodavanti et al., 2014; Hüffer and Hofmann, 2016; Bour et al., 2020; Gigault et al., 2021). Chemical similarity causes MP and NP to work as scavengers of toxic substances (Hüffer and Hofmann, 2016). Plastic micro- and nanoparticles can accumulate chemicals and serve as a carrier for long-range transport (Li et al., 2018; Kiran et al., 2022). Besides the POPs mentioned above, such substances as antibiotics or metals, e.g. Cr, Co, Ni, Cu, Zn, Cd and Pb can be accumulated and transported by micro- and nanoplastics (Holmes et al., 2012; Turner and Holmes, 2015; Li et al., 2018; Campanale et al., 2020).

**Figure 2:**
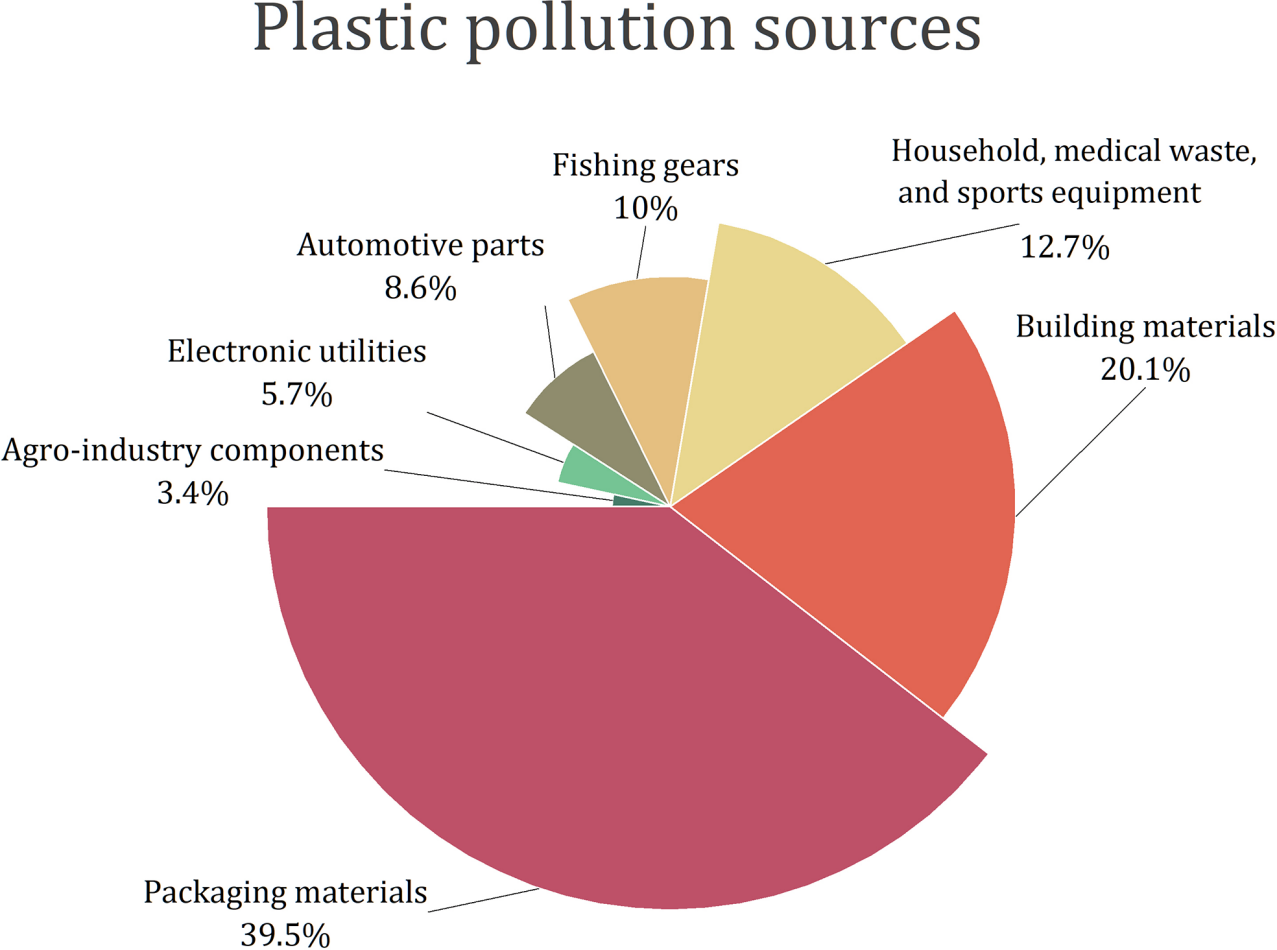
Plastic pollution sources (Horton et al., 2017; Pinto Da Costa et al., 2020; Kiran et al., 2022; Law and Narayan, 2022).

Plastics are used in virtually all commercial and industrial sectors (**Figure 2**).

Detection of MP and NP in plants or environmental samples is complex and challenging to perform. The analytical procedures allowing for the identification of the size, type, size (Caputo et al., 2021), and quantity of plastic pollutants depend strongly on the type of plant and chemical composition of plastic particles. Usually, the biological material before analysis must be pre-treated to eliminate organic matter, salts, or minerals that affect the detection of MP and NP (Zhang et al., 2022). Plastic particles can also be extracted from the sample (Li et al., 2021a). The most popular techniques are based on fluorescence microscopy (Maes et al., 2017). Also, techniques based on light scattering, such as dynamic light scattering (DLS) (Caputo et al., 2021) and multiangle light scattering (MALS), are popular (Correia and Loeschner, 2018). Plastics can also be detected based on their chemical composition. The best results of such way of analysis give infrared (Hernandez et al., 2019) and Raman spectroscopy (Fang et al., 2020), NMR, double shot pyrolysis - gas chromatography/mass spectrometry GC-MS (py-GC/MS) (Li et al., 2021a), thermal desorption proton-transfer-reaction mass spectrometry (TD-PTR-MS) (Materić et al., 2022) and energy-dispersive X-ray spectroscopy (EDX). Besides, single particle inductively coupled plasma mass spectrometry (spICP-MS) can be helpful in the detection of MP and NP (Jiménez-Lamana et al., 2020). Most importantly, no universal method for detecting plastics in biological and environmental materials exists. Each procedure requires testing and validation before it is used for plastic determination. However, it is worth emphasizing that research on this topic is still ongoing.

**Figure 3:**
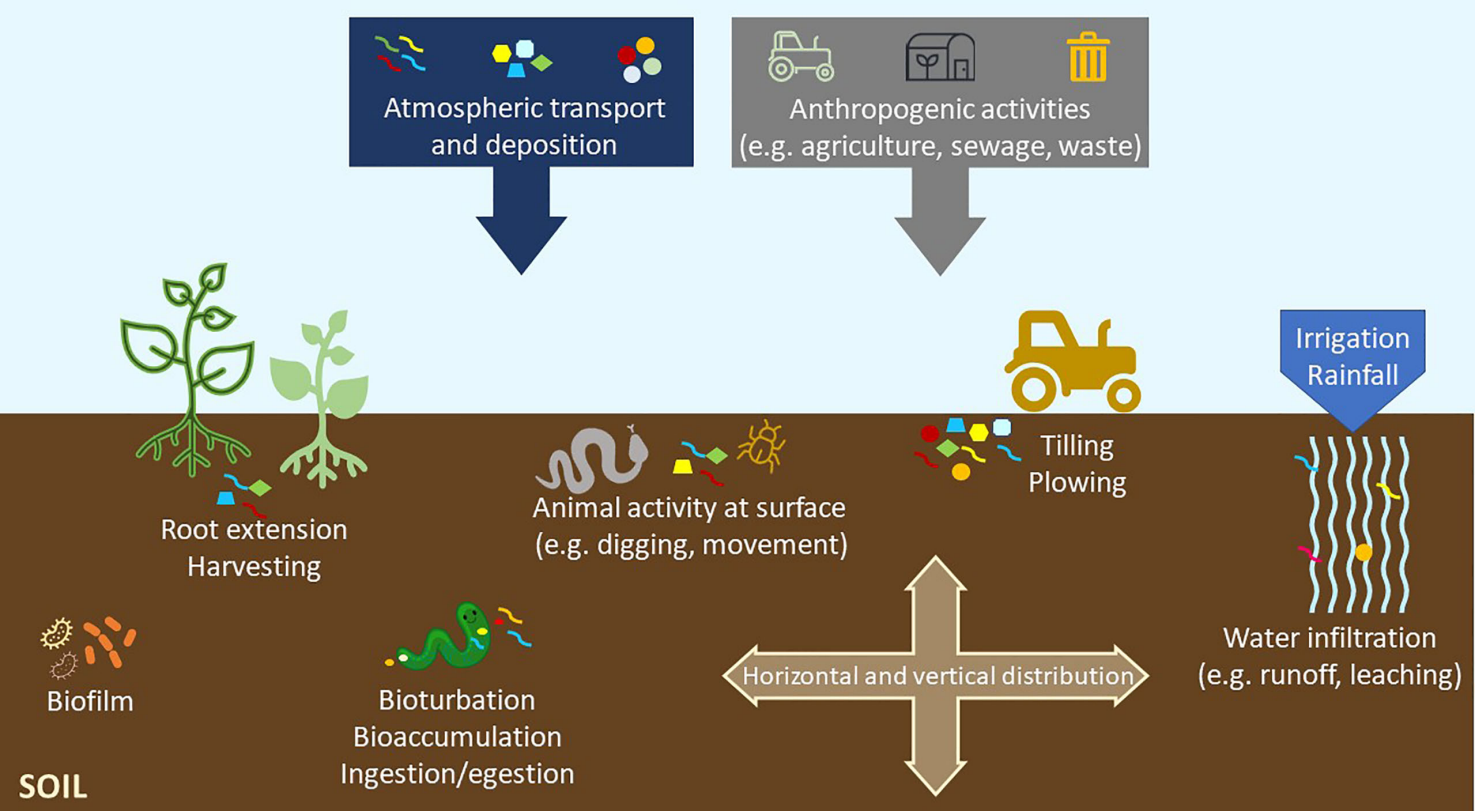
Sources of plastics in the terrestrial environment and factors that influence their vertical and horizontal distribution in the soil.

We often deal with two ways of introducing this pollutant into the environment - the primary or secondary MP and NP. The former is obtained in this form and used as, e.g. a carrier or abrasive material in cosmetics, toothpaste, detergents, personal care products, abrasive cleaning agents, plastic powder for moulding, and synthetic clothing, paints, electronics, etc. (Birch et al., 2020; Wang et al., 2020; Kihara et al., 2021; Kiran et al., 2022). The second type of NP and MP is the one that is created from larger fragments by their disintegration and degradation, e.g. under the influence of sunlight, temperature, humidity and wind, which account for 70-80% of all plastic released into the environment (Lambert and Wagner, 2016; Kiran et al., 2022). Most plastic pollution comes from anthropogenic activities, such as tire wear run off from roads, packaging, building materials, fishing gears, automotive parts, electronic utilities and agro-industry components (Geyer et al., 2017; Horton et al., 2017; Pinto Da Costa et al., 2020; Kiran et al., 2022; Law and Narayan, 2022; Castan et al., 2021; Huang et al., 2022), as well as from atmospheric transport (e.g. wind drift) (**Figure 3**). Agricultural activities perform a major role in introducing plastics into the soil through many ways, such as plastic mulching (which usually are not retrieved from the fields after its usage), use of plastic contaminated biosolids/sewage sludge for soil conditioning and use of plastic-containing wastewaters for irrigation (Liu et al., 2021; Castan et al., 2021; Huang et al., 2022). Once in the soil, plastics may be further transformed (through biodegradation and non-biodegradation processes, as explained previously) and transported and distributed in this compartment both horizontally and vertically. Many processes may contribute to such distribution in the soil (**Figure 3**). At the surface, activities of animals (e.g. digging, movement of animals on the surface) and agricultural tilling and harvesting may promote their horizontal distribution (Liu et al., 2021; Huang et al., 2021; Huang et al., 2022). Also, resuspension/remobilization (caused for example by wind or agricultural activities) may further contribute for their transport across soil surface. Vertically, the plastic particles may migrate into deeper soil layers through water’s infiltration processes, movement across soil cracks or spaces/holes in the soil that were created by plant roots elongation or edaphic organisms or through being transported by edaphic organisms (either bioaccumulated in the body or adsorbed into the skin), among others (**Figure 3**; Rillig et al., 2017; Heinze et al., 2021; Huang et al., 2021; Huang et al., 2022). Adding to these environmental factors, intrinsic properties of NP and of the soil matrices also influences their mobility in the soil (Wu et al., 2020; Brewer et al., 2021). Namely, plastics size, polymer type and shape, may determine their transport across the soil. As an example, O’Connor and collaborators reported that at similar sizes, polyethylene plastic particles tended to be transported more easily than those made of polypropylene (O’Connor et al., 2019). Soil physical and chemical properties, such as pH, ionic strength, ionic composition, also influence the transport of NP. Corroborating this, Wu and collaborators, revealed that the retention of polystyrene nanoplastic particles (PS NP) in the soil was negatively correlated with pH and ionic strength, while the presence of iron and aluminium oxides was positively correlated with the retention of those NP in the soil (Wu et al., 2020).

Plastic particles were detected both in marine, freshwater and soil environments (Allen et al., 2022; Lehner et al., 2019; Rai et al., 2021; Huang et al., 2021). They were found in the soils in various regions of the world e.g. in Australia, China, Chile, Germany, Switzerland (He et al., 2018; Schmid et al., 2021; Prata et al., 2021; Lehner et al., 2019). Moreover, NP and MP were noted not only in industrious regions but also in home gardens, farmlands and agricultural lands. Notably, MP and NP entering the environment can be harmful to living organisms (Prata et al., 2021). It was presented that NP may cause changes in plant organisms, e.g. disturbing growth, germination, genetics, physiology, morphology, photosynthesis and uptake of nutrients (described in more detail in section 2) (Xiao et al., 2022; Gong et al., 2022; Wang et al., 2022; Lian et al., 2020; Lian et al., 2022). Studies on animals showed that NP and MP might influence such trait as survival, morphology, behaviour, histopathology, development and reproduction, and in consequences population dynamic (An et al., 2021; Venâncio et al., 2022; Liu et al., 2022a; Huang et al., 2021). In addition, plastic particles by themselves and chemicals released into the environment from plastic waste may threaten plants and animals. For example, styrene or bisphenol-A released from plastic waste strongly affects marine organisms’ reproduction and the hormonal balance of animals, including terrestrial animals (Campanale et al., 2020). Moreover, the plastic particles may enter human bodies *via* three pathways: ingestion, inhalation and absorption by the skin (Prata et al., 2020). Among others, NP and MP may be transferred through food chains, e.g. from edible plants to humans. It was suggested that various types of NP constitute the most serious food and drinking water safety concerns (Pironti et al., 2021; Yee et al., 2021; UNEP, 2016). It was revealed that plastic particles may eventually enter the human bloodstream (Leslie et al., 2022) and may possibly affect human health (Lehner et al., 2019). Research conducted on human cell lines showed that MP and NP can induce apoptosis, oxidative stress, inflammation processes and gut microbiota dysbiosis of the cells (Yee et al., 2021).

There is increasing concern on the sufficient supply of safe food products that would meet the demands of growing human population. That is one of the reasons for rising interest in NP effect in plants within the scientific community, with four articles published in 2018 and already over eighty articles published in 2022 (Scopus database search with terms “nanoplastic*” AND “plant*”, date of access: 19.09.2022). The aim of present work is comprehensive revision of the accessible literature on the topic of plants response to NP. Special emphasis is put on plants oxidative response, which on the one hand is recognized as the marker of stress intensity and on the other hand, as an important element of stress signalling network.

## The impact of nanoplastic on plants

### Uptake and accumulation

**Figure 4:**
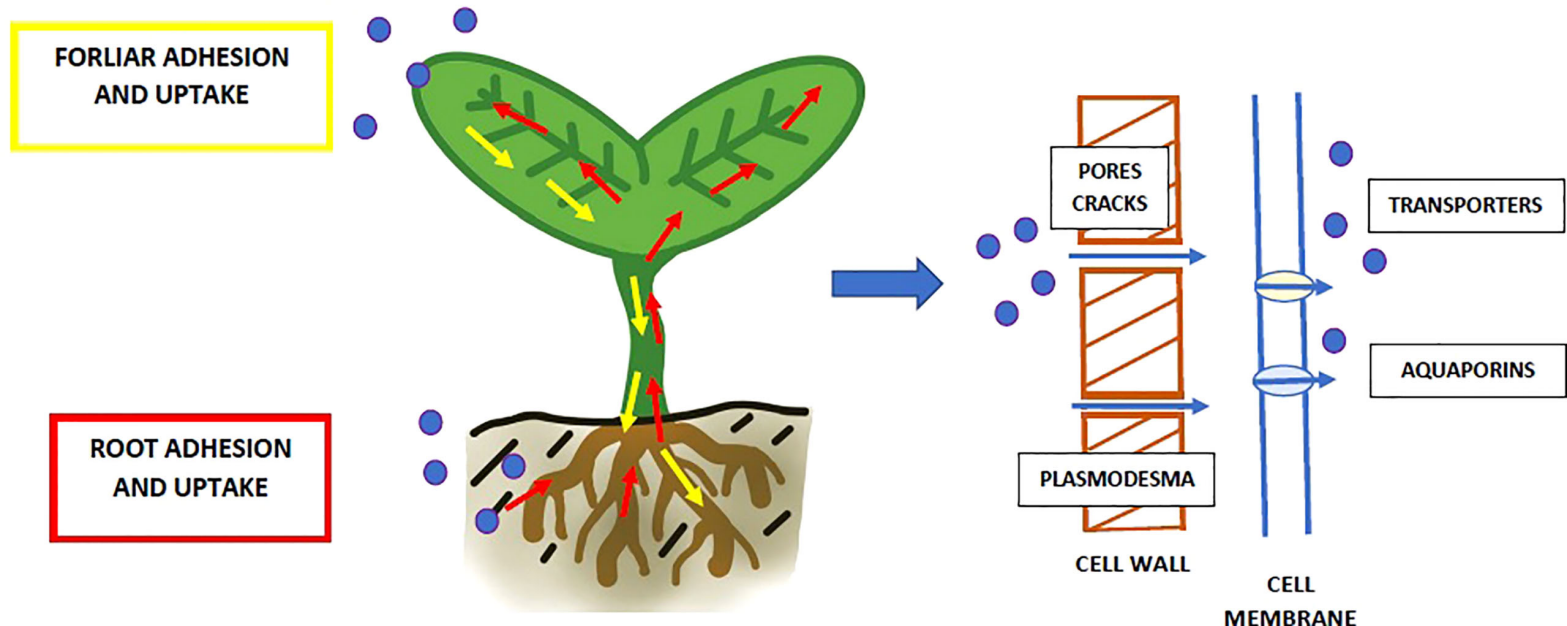
Uptake NP (blue dots) from the surrounded environment and transport through the plant organisms. Plastic particles may enter by roots (marked with red arrows) and leaves (marked with yellow arrows). The NP may enter the cell through pores and cracks in the cell wall and further through plasmodesma or transporters/aquaporins in the cell membrane.

Despite the increasing awareness of the risks associated with NP contamination of the environment, the number of studies on their impact on plants is still limited. The results of laboratory studies show that NP can be effectively taken up by the plants both *via* roots (Zhou et al., 2021; Sun et al., 2020; Zhou et al., 2021; Bosker et al., 2019) and leaves (Lian et al., 2021; Sun et al., 2021) (**Figure 4**). Afterwards, NP can be accumulated in inner tissues and migrated to other parts of the organisms. First of all, plastic particles adhere to the plants’ surface (Nolte et al., 2017; Xiao et al., 2022; Taylor et al., 2020). This process depends mainly on the surface chemistry of the structures (Nolte et al., 2017; Xiao et al., 2022). Research revealed that positively charged NP adhered significantly more to the plant surfaces than the negatively charged ones (Xiao et al., 2022; Sun et al., 2021). The cause is electrostatic attraction to the negatively charged cell wall. However, the situation looks differently when internalisation is taken into consideration. Contrary to the adhesion, positively charged NP entered the plant tissues at a lower level than negatively charged nanoplastics (Sun et al., 2020).

There are suggested several mechanisms of interaction between plants and plastic particles (Azeem et al., 2021; Mateos-Cárdenas et al., 2021). The NP and MP may adhere externally onto plant tissues. It concern mostly particles bigger than 200 nm (Mateos-Cárdenas et al., 2021). The cell wall is the first physical barrier against entering of foreign substances, including NP and MP, into plant organisms (Milewska-Hendel et al, 2019). It prevents penetration of particles larger than cell wall pores. However, some studies revealed that NP bigger than cell wall pores might internalise through cracks in the wall (Li et al., 2020). When NP adhere to the surface they may be just adsorbed on it and may be entrapped on surface structures.

On the other hand, the NP may be internal taken up by plants, what depends mostly on their size and charge (what was mentioned above). The potential pathways for nanoparticles to enter plant cells can be plasmodesmata channels, endocytosis, ion transporters and aquaporins (Khan et al., 2019; Zhou et al., 2021). As mentioned above, there are two pathways of exposure: roots or foliar foliage (**Figure 4**). NP taken up by roots’ hair or tip are transported to other organs, such as stems and leaves (Giorgetti et al., 2020; Sun et al., 2020; Xiao et al., 2022). One of the primary influences on this movement is the pull of transpiration (Li et al., 2020). In the case of foliar application, it was revealed that NP which adhere to leaves might be taken up through the stomatal opening (Sun et al., 2021; Lian et al., 2021). Afterwards, they are transported by phloem (Lian et al., 2021). NP enter vasculature and are then translocated downward to the roots in the vascular bundle. It was shown that plastic particles might be effectively accumulated in plant organisms. They were detected in epidermal cells, apoplast and xylem, as well as along the cell walls (Sun et al., 2020; Liu et al., 2022b; Zhou et al., 2021). The plastic nanoparticles (50 nm PS NP) were observed also inside the root cells, in cytoplasm and vacuoles (Spanò et al., 2022).

An uptake, translocation and accumulation depend on several factors. One of the most important is the size (Liu et al., 2022b). Smaller NP are better transported than larger ones. Similarly, aggregation may change their movement speed, mainly because of altering their size (Sun et al., 2021). The root NP uptake might be modulated by root exudates – low- and high molecular organic compounds, which were shown e.g. to interact with arbuscular mycorrhizal fungi (AMF) and plant growth promoting bacteria (PGPB), mitigate draught and metal stress and facilitate phosphorus (P) and iron (Fe) uptake (Chai and Schachtman, 2022). The compounds secreted to the soil by plants and microorganisms are referred also as extracellular polymeric substances (EPS). It has been shown that interaction of PS NP with EPS results in NP aggregation and significant increase in their mean hydrodynamic diameter (MHD), possibly affecting also their uptake (Giri and Mukherjee, 2022).

In the case of foliar application, after some time, aquaporin channels begin to shut down in response to NP stresses, which may cause a reduction in the uptake of NP (Zhou et al., 2021). In addition, root-to-shoot NP translocation can be altered by changes in transpiration pull in plants (Li et al., 2020). Moreover, upwards transport depends on exposure time (Li et al., 2021a). Furthermore, both up and down movement may also be changed by such factors as NP surface charge and shape, presence of other substances, kind of media for plant growth and plant species (Sun et al., 2020; Zhou et al., 2021; Taylor et al., 2020).

### Phytotoxicity

Nanoplastic has varying effects on plants growth. For instance, in wheat low PS NP concentration (0.01-10 mg/l; size between 151 and 870 nm) boosted the growth as reflected by increase in the roots and shoots biomass and relative root elongation (Lian et al., 2020). Simultaneously, a decrease in the shoot/root ratio has been observed (Lian et al., 2020). However, most of the reports indicate that NP at low concentration do not affect plants growth, while higher concentrations lead to growth reduction, as observed in *Arabidopsis* in response to 300 and 1000 mg/l of 70 nm PS-NH_2_ (cultivation in soil) or 100, 500 and 1000 mg/l of 70 nm PS-NH_2_ and 55 nm PS-SO_3_H (cultivation in MS medium), broad bean seedlings in response to 100 mg/l of 100 nm PS NP, onion seedlings in response to 100 and 1000 mg/l of 50 nm PS PNs or rice seedlings in response to 50 and 100 mg/l of 20 nm PS NPs (Giorgetti et al., 2020; Jiang et al., 2019; Sun et al., 2020; Zhou et al., 2021). In the cited reports the NP were applied in the growing medium. In the case of foliar application, even lower concentrations negatively affected plants growth. Treatment of lettuce plants for 21 days with 100 nm PS NP at the concentrations of 0.1 and 1 mg/l resulted in decrease in plants height, leaf area and dry weight (Lian et al., 2021). The effects depend also on the diameter of the particles. In cucumber plants only 100 nm PS NP reduced roots length and thickness, while 300, 500 and 700 nm PS NP did not affect these parameters in a significant way (Li et al., 2021c). In the majority of the studies NP are applied in the form of polystyrene particles. However, the individual study using polymethyl methacrylate (PMMA) nanoparticles confirmed the negative impact of NPs on plants growth. In this study a concentration dependent decrease in the growth in response to 130 nm PMMA NP has been observed in lettuce (Yildiztugay et al., 2022).

The NPs-driven growth inhibition might be at least partially dependent on alterations in mineral homeostasis. In lettuce, foliar exposure to 90 nm PS NP resulted in lower Fe and Zn levels in the leaves and lower Fe, Zn, Cu and Mn levels in the roots (Lian et al., 2021). In turn treatment with 130 nm PMMA NP resulted in reduced content of Fe, Zn, Cu, Mn, Ca, Mo, K, P and B (Yildiztugay et al., 2022). Similarly, in cucumber,14-days long treatment with PS NP (100, 300, 500, and 700 nm) led to decrease in the content of Fe, Ca and Mg (Li et al., 2021c). The described studies provide evidence for the NP interaction with nutrients uptake. Due to the limited literature on the topic, it would be difficult to propose a possible mechanism standing behind the hampered uptake of specific elements. It is possible that, similarly to e.g. metals, NP compete for the sites in specific transporters or negatively affect transporters expression/activity.

Hampered growth might also results from disturbances in photosynthesis and respiration leading to reduced energy supply. Indeed, exposure of rice plants to neutrally charged nanoparticles (PS) as well as negatively (PS-COOH) and positively (PS-NH_2_) charged NPs (size about 50 nm) resulted in decrease in net photosynthesis values, wherein the strongest effects were observed in response to PS-NH_2_. Additionally, the positively charge PS NP caused decrease in the level of chlorophyll a and b (Wang et al., 2022). In lettuce 130 nm PMMA NP negatively affected maximum quantum yield of photosystem II (PSII) and potential photochemical efficiency (Yildiztugay et al., 2022).

The NP taken up by plants adversely affect their genetic material reflected e.g. by increase in micronuclei, C-metaphases and sticky chromosomes formation and impeded cell division (Giorgetti et al., 2020; Jiang et al., 2019; Spanò et al., 2022). For example, in meristems of onion, an inhibitory effect of 50 nm PS NP on the mitotic index (MI) at 100 and 1000 mg/l was observed. The MI in meristems decreased by 34.4% at 100 mg/l and by 41.9% at 10, 00 mg/l. It was found that the frequency of abnormal metaphases was 18.5%, while abnormal anaphases and telophases - 12.7%, when the NP at concentration 10 mg/l were applied. It is worth noting that the number of cytological anomalies did not increase with increasing NP concentrations (Giorgetti et al., 2020). The MI of rice root meristems under exposure to 50 nm PS NP decreased by 34.85% relative to control at the highest concentration (1000 mg/l) (Spanò et al., 2022). Similarly, reduction of MI in response to 100 nm PS NP were observed in castor bean (Jiang et al., 2019). Therefore, it can be assumed that NP exhibit cytotoxic effects and that the level of MI decrease depends on the concentration of nanoplastic and the plant species studied.

Nanoplastic is also known to lead to the formation of mitosis abnormalities. For instance, previous studies of onion have found an increase in micronuclei formation under exposure to 50 nm PS NP at the highest applied concentration of 1000 mg/l (Giorgetti et al., 2020). Exposure of castor bean to 100 nm PS NP also showed increased formation of micronuclei at the highest applied concentrations (100 mg/l) (Jiang et al., 2019). In addition, exposure to NP results in formation of C-metaphases, delayed metaphases, and sticky chromosomes detected during mitosis metaphase. Increased C-metaphases were observed at 100 and 1000 mg/l of 20-200 (mainly 100) nm PS NP and constituted the most common abnormality of mitosis in rice plants exposed to nanoplastic (Spanò et al., 2022). Treatment with NP also led to the formation of lagging chromosomes observed in anaphase and telophase. In total, cytological abnormalities of mitosis under NP exposure at the highest applied concentrations have been shown to reach about 30% in onion and 40% in rice, which is a large deviation in relation to the control plants (Giorgetti et al., 2020; Spanò et al., 2022).

Exposure to NP modulates genes expression on the transcriptomic level. Study carried out on *Arabidopsis thaliana* showed that seven weeks long exposure to 70 nm PS-NH_2_ PS NP leads to up-regulation of genes associated with the secondary metabolism and attenuated levels of transcripts associated with ROS metabolism and stress response (Sun et al., 2020). In wheat 100 nm PS NP affected expression of genes associated with carbon and amino acid metabolism. In addition, alteration in the expression of genes related to cellular signalling were reported (Lian et al., 2022). In turn in rice, the genes related to antioxidant system, stress response, regulation of cell cycle and RNA and polysaccharides metabolism were differentially regulated under 50 nm PS NP treatment (Wang et al., 2022). The studies on duckweed revealed that even low 126 nm PS NP concentrations (0.015 µg/l) induced changes in the expression of genes e.g. associated with organelle envelope and inner membrane, oxidation-reduction processes, calcium signalling and tetrapyrrole binding. Exposure to higher concentrations (5 µg/l) resulted in up-regulation of genes associated with the response to stimuli and down-regulation of genes related to amino acid and carbohydrates transport and acetylo-CoA and thioester metabolism (Xiao et al., 2022).

The cited above studies show that NP may be effectively absorbed by plants and that their accumulation leads to the development of typical stress symptoms: reduced growth, alerted mineral homeostasis, decreased photosynthesis efficiency, genotoxic effects and modulation of genes expression, including down-regulation of basic metabolism associated genes and up-regulation of genes involved in defence mechanisms. Thus, it is evidenced that NP constitute an abiotic stress factor, which has been relatively recently introduced in the environment.

## Plants oxidative response

### Reactive oxygen species effects in plant cells

Plants oxidative status is dependent, on the one hand, on the production of reactive oxygen species (ROS) and, on the other hand, on the efficiency of their scavenging by the antioxidant system. The major species of reactive oxygen include superoxide anion (O_2_^·^), hydrogen peroxide (H_2_O_2_), hydroxyl radical (·OH) and singlet oxygen (^1^O_2_). These species differ in their origin, lifetime, reactivity and biological impact. Superoxide anion is recognized as the primary ROS, produced mainly in electron transport chains in chloroplasts and mitochondria and by a membrane bound enzyme, NADPH oxidase (NOX), also referred to as RBOH (respiratory burst oxidase homologs). This ROS is highly reactive but is readily converted to moderately reactive hydrogen peroxide (Fichman and Mittler, 2020; Farnese et al., 2016; Demidchik, 2015). Due to its relatively long lifetime (>1 ms) and ability to pass through specific aquaporins (Bienert et al., 2007; Rodrigues et al., 2017), H_2_O_2_ can travel a considerable distance and react, mainly with proteins, in various cellular compartments. In turn, hydroxyl radical is the least stable ROS with half lifetime measured in ns. It is the most reactive species and can cause considerable damage near the site of its formation. Singlet oxygen is produced mainly by chloroplasts through the transfer of energy from the triplet state of chlorophyll to oxygen. This ROS is characterized by relatively high reactivity and is the main source of oxidative damage of the photosystems in chloroplasts (Das and Roychoudhury, 2014; Dumanović et al., 2021). Reactive oxygen species are quenched by enzymatic and non-enzymatic antioxidants. The main antioxidant enzymes include superoxide dismutase (SOD), catalase (CAT) and peroxidases (POX). The groups of non-enzymatic antioxidants include e.g. ascorbic acid (ASC), glutathione (GSH), tocopherol, carotenoids and phenolic compounds (Dumanović et al., 2021).

Initially ROS were perceived solely as damaging agents, which cause oxidation and impairment of biomolecules including lipids, proteins and nucleic acids. Nowadays it is commonly acknowledged that certain ROS level is indispensable for proper cell functioning as they constitute important signalling elements and are engaged in regulation of gene expression, developmental processes and stress response. An important mode of their action is dependent on the oxidation of cysteine residues leading to the formation of disulphide bridges affecting protein structure and functioning. Oxidation of cysteine regulates the activity of various proteins including glyceraldehyde-3-phosphate dehydrogenase (GAPDH), mitogen-activated protein kinases (MAPK) and H_2_O_2_ sensing HPCA1 protein (Mittler, 2017; Castro et al., 2021). It is worth highlighting that some proteins, such as thioredoxins (TRX) and glutaredoxins (GRX), are especially prone to oxidation of cysteine residues and can act as redox signal transmitters. These molecules can mediate the oxidation and reduction state of cysteines in other proteins and thus work as redox switches (Martí et al., 2020).

In addition to the direct impact on proteins, ROS can modulate signalling network through interaction with other signalling elements such as calcium ions (Ca^2+^), reactive nitrogen species (RNS), reactive sulphur species (RSS) plant hormones, mitogen activated protein kinases, transcription factors and other signalling associated proteins (Castro et al., 2021; Farnese et al., 2016). It is postulated that various signalling pathway are integrated in signalling hubs and that NADPH oxidase (RBOH) may constitute such signalling integration and coordination site. This membrane-bond enzyme can be regulated by calcium fluctuations, direct phosphorylation and binding of phosphatidic acid. In addition, an increasing amount of evidence shows that NADPH oxidase activity can be affected by S-nitrosation and presulfidation, connecting it with the RNS and RSS signalling pathways. Its activation leads to increased production of O_2_^·^, which is thereafter converted to the main ROS signalling molecule - H_2_O_2_. RBOH proteins are key elements in propagation of ROS waves, referred to as rapid, long-distance and autopropagating signals. The sequence of ROS wave generation includes accumulation of H_2_O_2_ in apoplast and its sensing by HPCA1 protein resulting in increased influx of Ca^2+^ into the cytosol. This in turn leads to activation of NADPH oxidases and amplification of ROS signal (Castro et al., 2021; Fichman and Mittler, 2020).

It is postulated that also the products of ROS-dependent oxidation can constitute signalling or gene regulatory elements. For instance, oxylipins, which can be formed as a result of lipid peroxidation, affect expression of numerous genes in *Arabidopsis* plants. The most frequent oxidative modification of ribonucleic acids, 8-hydroxyguanosine (8-OHG), is formed in a selective manner in specific transcripts, leading to their hampered translation and attenuate levels of certain proteins. This process is likely engaged in pathogenesis of neurodegenerative diseases in animals and in alleviation of seed dormancy in plants. In turn, small peptides derived from protein oxidation could, in addition to general information on the occurrence of oxidative stress, also transmit the information on its intensity, the type of over-produced species and stress localization (Chmielowska-Bąk et al., 2015).

As described above, ROS can modulate signalling through direct interaction with proteins (formation of disulphide bonds) including redox transmitters (e.g. TRX, GRX), interaction with other signalling elements (e.g. RNS, RSS, Ca^2+^, MAPK) or possible generation of further signalling or gene regulatory elements (oxidized peptides, oxylipins, 8-OHG enriched transcripts). On the other hand, ROS excess might lead to oxidative damage of molecules and thus exhibits deleterious effects. Enhancement of ROS production is a common plant response to various stress conditions (Fichman and Mittler, 2020; Farnese et al., 2016; Demidchik, 2015). However, it is quite difficult to elucidate if stress-dependent ROS accumulation is a symptom of oxidative stress or signalling and/or regulatory response.

### Oxidative response of plants to nanoplastic

An increasing number of reports show that, similarly to other stress factors, also NP induce an oxidative response in plants (summarized in **Table 1**). The studies show that even short-term exposure to NP can result in the accumulation of ROS. In onion seedling, treatment for 72 h with PS NP at the concentration of 1000 mg/l resulted in a significant increase in the H_2_O_2_ level. On the other hand, exposure to lower concentrations, 10 and 50 mg/l, had no significant effect. The results were confirmed by microscopic detection using Amplex Ultra Red probe showing concentration-dependent accumulation of H_2_O_2_ in the root tissues of the treated seedlings (Giorgetti et al., 2020). The NP effects might depend on their surface charge. In *Arabidopsis*, an increase in H_2_O_2_ and O_2_^·^ level in response to PS NP was noted, wherein the positively charged NP (PS-NH_2_) induced stronger H_2_O_2_ accumulation than the negatively charged ones (PS-SO_3_H) (Sun et al., 2020). Similar results were obtained in dandelion and duckweed - in the studies, both positively as well as negatively charged NP caused an increase in the ROS level. However, the response was more pronounced in the case of PS-NH_2_ (Gao et al., 2022; Xiao et al., 2022).

**Table 1:** Summery of plant oxidative response to NP (≤100 nm); PS, polystyrene; PMMA, polymethyl methyacrylate; MD, malondialdehyde (marker of lipid peroxidation); TBARS, thiobarbituric acid reactive substances (marker of lipid peroxidation); APX, ascorbic peroxidase; CAT, catalase; POX, peroxidases; SOD, superoxide dismutase.

Plant species	NPs type, size and concentration	Treatment duration	NPs effect	References
castor bean (*Vicia faba*)	PS; 100 nm; 10, 50 and 100 mg/l	48 h	concentration dependent changes in lipid peroxidation and the activity of antioxidant enzymes	(Jiang et al., 2019)
wheat (*Triticum aestivum*)	PS; on average 87 nm; 10 mg/L	21 days	↑ lipid peroxidation	(Lian et al., 2020)
onion (*Allium cepa*)	PS; 50 nm; 10, 100 and 1000 mg/L	72 h	↑ lipid peroxidation and hydrogen peroxide accumulation	(Giorgetti et al., 2020)
arabidopsis (*Arabidopsis thaliana*)	PS; PS-SO_3_H: 55 nm, PS-NH_2_: 71 nm 300 and 1000 mg/L	7 days, 10 days, 7 weeks	↑ hydrogen peroxide and superoxide anion level modified genes expression	(Sun et al., 2020)
rice (*Oryza sativa*)	PS; 19 nm; 10, 50 and 100 mg/l;	16 days	↑ activity of antioxidant enzymes	(Zhou et al., 2021)
rice (*Oryza sativa*)	PS; 50 nm; 100 and 1000 mg/l	4 days	roots: ↓ hydrogen peroxide and TBARS levels ↑ APX and SOD activity Shoots: ↓ TBARS levels ↑ APX, POX and CAT activity	(Spanò et al., 2022)
rice (*Oryza sativa*)	PS, PS-COOH, PS-NH_2_ 50 nm 50 mg/l	7 days	Alerted activity of POD, SOD and CAT ↑ TBARS in response to PS-NH_2_ ↓TBRAS in response to PS-COOH	(Wang et al., 2022)
cucumber (*Cucumis sativus*)	PS; 100, 300, 500, 700 nm; 50 mg/l	14 and 65 days	particle size dependent changes in lipid peroxidation and vitamin C level	(Li et al., 2021c)
lettuce (*Lactuca sativa*)	PS (foliar application); 0.1 and 1 mg/l; 100 nm	30 days	↓ decreased antioxidant activity	(Lian et al., 2021)
lettuce (*Lactuca sativa*)	PS; 100 nm 50 mg/l	21 days	↑ MDA level ↑ SOD activity	(Gong et al., 2022)
dandelion (*Taraxacum asiaticum*)	PS: PS-COOH, PS-NH_2_ 80 nm 1, 5, 10 mg/l	7 days	↑ hydrogen peroxide and superoxide anion level ↑ SOD and CAT activity ↑ ASC and GSH ↓ polyphenols, flavonoids	(Gao et al., 2022)
duckweed (*Lemna minor*)	PS: PS-SO_3_H, PS-NH_2_ Approx. 100 nm 2-50 µg/l	3 days	↑ hydrogen peroxide, superoxide anion and TBARS level	(Xiao et al., 2022)

Accumulation of ROS leads to the oxidation and damage of cellular elements, wherein lipid peroxidation is probably the most frequently assessed oxidative stress marker. The commonly measured products of lipid peroxidation include malondialdehyde (MDA) and thiobarbituric acid reactive substances (TBARS). Independent studies on onion and broad bean seedlings treated for a short time with PS NP, revealed concentration-dependent changes in lipid peroxidation. The lower concentrations (10 mg/l) resulted in a decrease in TBARS/MDA levels, while the highest applied concentrations (100 mg/l in the case of broad bean and 1000 mg/l in the case of onion) led to intensified lipid peroxidation (Giorgetti et al., 2020; Jiang et al., 2019). Interestingly, in the case of broad bean, the same study showed that treatment with microplastic (MP) of 5 µm diameter did not affect MDA levels (Jiang et al., 2019). Size-dependent effects of NP on lipid peroxidation were also shown in cucumber. In response to a 14-day exposure, an increase in MDA level has been noted only in the case of 300 and 500 nm PS NP, while no effect has been observed in the case of 100 and 700 nm PS NP (Li et al., 2021c). In turn, study on rice indicated that the intensity of lipid peroxidation is dependent on the NP charge. In the roots, neutrally, negatively and positively charged NP augmented lipid peroxidation. However, the most significant effect was observed in the case of neutral PS NP. In turn, in the shoots, neutrally charged NP had no influence on lipid peroxidation, positively charged led to a significant TBARS accumulation, while treatment with negatively charged NP resulted in alleviated TBARS levels in relation to the control (Wang et al., 2022). Similarly, in duckweed the effect of PS- NH_2_ on lipid peroxidation was stronger and induced by lower concentrations than the effect of PS-SO_3_H (Xiao et al., 2022). The symptoms of oxidative stress were observed not solely by the application of PS NP but also in response to PMMA. In this case, application of PMMA particles to hydroponically grown lettuce resulted in accumulation of H_2_O_2_ and TBARS. However, it should be noted that in this case the particles were slightly bigger than in the other cited studies, with average diameter of 131 nm (Yildiztugay et al., 2022).

The majority of the described studies show NP-dependent increase in ROS level and intensified lipid peroxidation. However, there are also individual reports presenting opposite findings. In the roots of young rice seedlings, reduced H_2_O_2_ levels in response to lower PS NP concentration (100 mg/l) were reported, while higher concentrations did not affect ROS accumulation. In addition, PS NP-dependent decrease in the TBARS levels was observed in seedlings roots and shoots. The authors propose that this effect could result from enhanced activity of the antioxidant system. Indeed, PS NP stimulated the activity of guaiacol peroxidase (POX), ascorbate peroxidase (APX) and superoxide dismutase (SOD) in the roots, while the activity of POX, APX and catalase (CAT) was induced in the shoots (Spanò et al., 2022). These results were confirmed by other studies on rice showing stimulation of peroxidases (POX), SOD and CAT in the roots in response to PS NP (Wang et al., 2022; Zhou et al., 2021). Similarly, in castor bean, treatment with PS NP resulted in enhanced activity of SOD and CAT, while POX showed increased activity by lower concentrations but a decrease in response to the higher concentration (Jiang et al., 2019). In dandelion, PS NP-dependent stimulation of SOD and CAT has been reported, wherein the response was the most prominent in the case of positively charged particles in relation to the neutrally and negatively charged ones (Gao et al., 2022). Exposure to PS NP can also modulate the level of non-enzymatic antioxidants. In cucumber, size-dependent effects on the accumulation of ascorbic acid (ASC) have been described - 100 nm PS NP causes an increase in the ASC level, whereas application of 500 nm PS NP resulted in the decrease in its level (Li et al., 2021c). PS NP-dependent increase in the level of ASC and glutathione (GSH) has been noted also in dandelion plants. On the other hand, exposure to PS NP led to a reduced content of polyphenols including flavonoids, which constitute a group of bioactive compounds with antioxidant activity (Gao et al., 2022).

In the environment, plants are frequently exposed to combined stress factors, which can further aggravate their negative impact. Recent study on lettuce focused on the combined action of FeNP and PS NP. The results showed that the nanoparticles form hetero-aggregates. Simultaneous application of Fe NP and PS NP also resulted in augmented Fe release and accumulation in plant tissues. In addition, an increase in the level of lipid peroxidation and augmented cell damage were noted in the treated plants (Gong et al., 2022).

The NP-dependent accumulation of ROS is convincingly evidenced. The extent of the reaction is dependent on various factors including NP concentration, size and charge. The ROS over-production is associated with intensified lipid peroxidation, which is a typical symptom of oxidative stress. In general, ROS accumulation might also lead to protein carbonylation and oxidation of nucleic acids (Das and Roychoudhury, 2014; Dumanović et al., 2021). To the best of our knowledge so far there is no information on NP driven modifications of proteins. However, there are reports showing the adverse impact of these particles on the genetic material reflected by an increase in the amount of micronuclei formation and/or cytological abnormalities and decreased mitotic index (Jiang et al., 2019; Giorgetti et al., 2020; Spanò et al., 2022). In addition, ROS might exert signalling and gene regulator role. Indeed, changes in gene expression in response to NP have been observed in *Arabidopsis*, rice, wheat and duckweed (Lian et al., 2022; Sun et al., 2020; Wang et al., 2022; Xiao et al., 2022). However, it is not known if the observed changes are dependent on ROS action.

Thus, it can be concluded that although the information on NP oxidative effects in plant is gradually increasing, there is still much to be examined. So far the reports are limited to few plant species: Arabidopsis, rice, wheat, onion, cucumber, lettuce and duckweed. In addition, the effects were studied mainly in response to long-term exposure and to one type of plastic - polystyrene. It is evidenced that NP induce ROS formation and peroxidation of lipids. However, there is no information on the oxidation of other cellular compounds including proteins and nucleic acids. In addition, NP-dependent ROS accumulation is examined in relation to their cytotoxicity, while little attention is given to the possible signalling and gene regulatory roles.

## Conclusions

**Figure 5:**
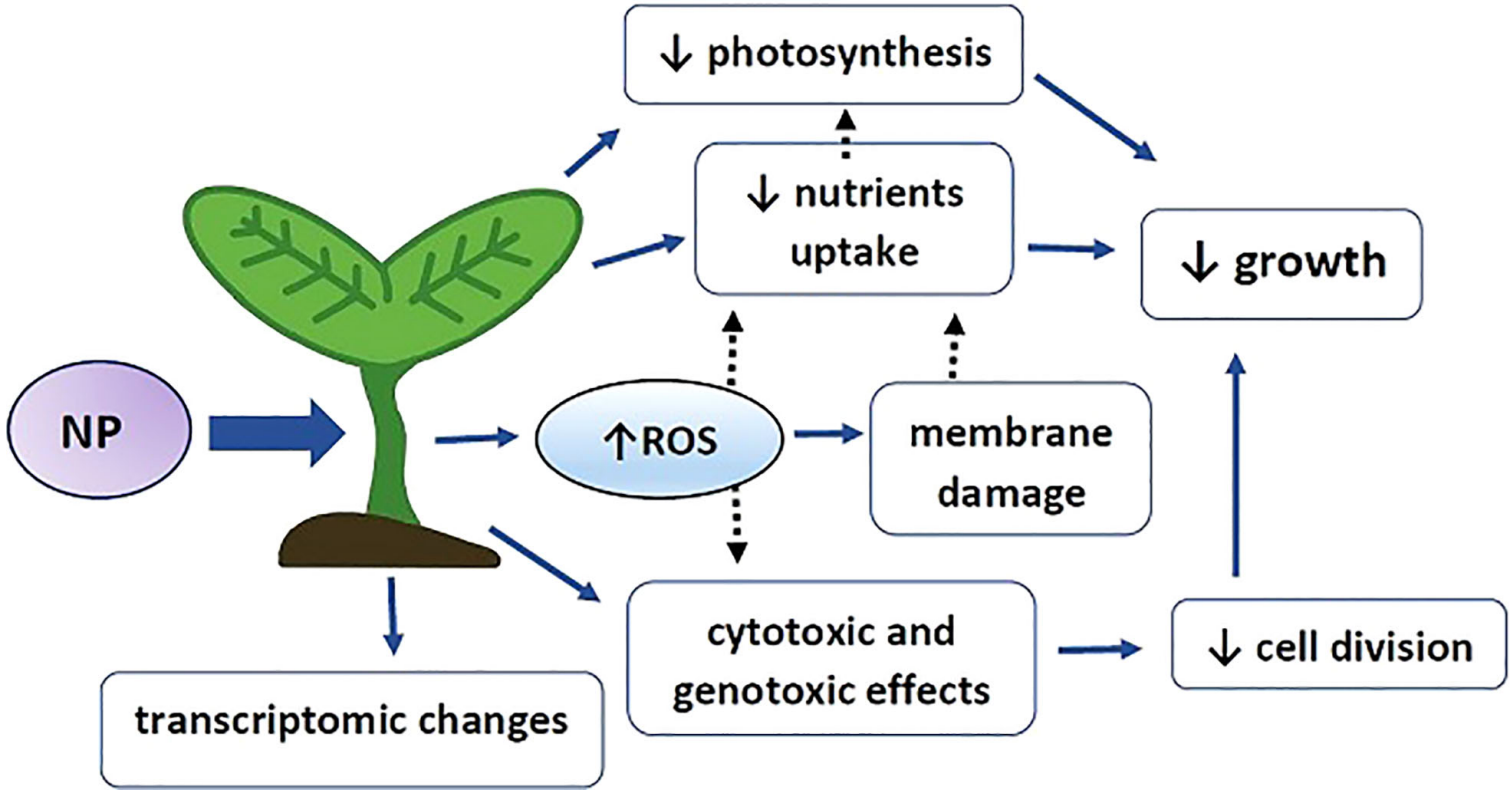
Graphical summary of NP impact on plants. NP – nanoplastic particles, ROS – reactive oxygen species, blue arrows – experimentally evidenced effects, black dotted arrow – possible interactions. Exposure to NP leads to increase in reactive oxygen species, hampered uptake of specific nutrients, decrease in photosynthesis efficiency and cyto- and genotoxic effects. ROS might mediate cyto- and genotoxic effect through direct interaction with genetic material and alert nutrient uptake through mediation of membrane damage. Decrease in the level of certain elements e.g. Fe and Mg might results in impaired chlorophyll synthesis and contribute to lower photosynthesis efficiency. Cyto- and genotoxic effects lead to decreased cell division. Alerted cell division, nutrient level and photosynthesis results in hampered plants growth. In addition, NP alert plants physiology through modulation of genes expression on the transcriptomic level.

An increasing amount of evidence shows a progressive contamination of the environment with plastic, including nanoplastic particles (NP). It can be predicted that the problem will aggravate with time due to high plastic production and simultaneous long degradation time. Recent studies on plants show that exposure to NP leads to the development of toxicity symptoms including growth inhibition, alterations in mineral homeostasis and photosynthesis efficiency, decreased cell division and genotoxic effects (summarized in **Figure 5**). A common plant response to abiotic stress factors, including environmental contaminants, is over-production of reactive oxygen species (ROS). Indeed, in most of the studies, exposure to NP also resulted in accumulation of superoxide anion and/or hydrogen peroxide, accompanied by intensified lipid peroxidation and stimulation of the antioxidant system. The published data indicated that the oxidative response is in general stronger in the case of positively charged and smaller plastic particles (when comparing MP to NP). However, the exact effects of NP-driven ROS, including their impact on the oxidation of proteins and nucleic acids and possible gene regulatory/signalling functions, still remain unexamined.

## Author contributions

AE-G and JC-B elaborated manuscript concept, all authors participated in manuscript writing and editing. All authors contributed to the article and approved the submitted version.

## Funding

The research on nanoplastic impact on plants carried out in the Department of Plant Ecophysiology is financed by statutory funding (JC-B) and by the National Science Centre, Poland, grant number UMO-2016/23/D/NZ8/01112 (AE-G). This work was supported in the scope of the project CESAM- UIDB/50017/2020 + UIDP/50017/2020 + LA/P/0094/2020, financed by national funds through the FCT/MEC (IL).

## Acknowledgments

We would like to thank the members of COST action CA20101 “Plastics monitoRIng detectiOn RemedIaTion recoverY” for fruitful discussion on the topic of nanoplastic contamination and impact on plants.

## Conflict of interest

The authors declare that the research was conducted in the absence of any commercial or financial relationships that could be construed as a potential conflict of interest.

## Publisher’s note

All claims expressed in this article are solely those of the authors and do not necessarily represent those of their affiliated organizations, or those of the publisher, the editors and the reviewers. Any product that may be evaluated in this article, or claim that may be made by its manufacturer, is not guaranteed or endorsed by the publisher.

## Abbreviations

NP, nanoplastic particles; MP, microplastic particles; PS, polystyrene; PMMA, polymethyl methacrylate; ROS, reactive oxygen species.
